# Platelet-like cells differentiated from adipose-derived mesenchymal stem cells inhibit acute inflammation of tendinopathy in rats

**DOI:** 10.1007/s00774-025-01647-2

**Published:** 2025-10-03

**Authors:** Akiko Torii, Yuichi Yamada, Yukako Ono-Uruga, Yuiko Sato, Yosuke Kaneko, Satoshi Nakamura, Takuji Iwamoto, Yumiko Matsubara, Morio Matsumoto, Masaya Nakamura, Kazuki Sato, Takeshi Miyamoto

**Affiliations:** 1 Department of Orthopedic Surgery, Keio University School of Medicine, 35 Shinano-Machi, Shinjuku-Ku, Tokyo, 160-8582 Japan; 2 Institute for Integrated Sports Medicine, Keio University School of Medicine, 35 Shinano-Machi, Shinjuku-Ku, Tokyo, 160-8582 Japan; 3 Center for Integrated Medical Research, Keio University School of Medicine, 35 Shinano-Machi, Shinjuku-Ku, Tokyo, 160-8582 Japan; 4 Department of Orthopedic Surgery, Kumamoto University, 1-1-1 Honjo, Chuo-Ku, Kumamoto, 860-8556 Japan

**Keywords:** Platelet-rich plasma, Adipose-derived mesenchymal stem cells, Inflammation, Tendinopathy

## Abstract

**Introduction:**

Tendinopathy, a disease that causes inflammation and pain and limits patients’ activities of daily living, is considered particularly important to treat during the acute inflammatory phase to prevent the transition to chronic degeneration. Recently, platelet-rich plasma (PRP) has been used to treat tendinopathy; however, it is not clear whether platelets themselves, which are the active component of PRP, could be effective in treating tendinopathy.

**Materials and Methods:**

We made rat Achilles tendinopathy models by incision of the calcaneal attachment and administrated platelet-like cells derived from adipose-derived mesenchymal stem cells (ASCL-PLCs) to the injury site and investigated the anti-inflammatory effect.

**Results:**

ASCL-PLCs significantly inhibits the inflammatory cytokine expression and inflammatory cell infiltration in acute tendonitis in a rat Achilles tendon injury model in vivo. Interestingly, we observed no xeno-reaction when human-derived ASCL-PLCs were administered to wild-type rats in vivo. Moreover, IL-6 expression and phosphorylation seen in NIH3T3 fibroblasts treated with IL-6 plus soluble IL-6 receptor were both significantly suppressed by ASCL-PLCs in vitro.

**Conclusion:**

ASCL-PLC has advantages over existing PRP therapies, including the ability to be cryopreserved after quality checks, and homogeneous populations of ASCL-PLCs can be prepared in large quantity. We conclude that in the future ASCL-PLCs may serve as an allogeneic transplant effective to treat tendinopathy.

**Supplementary Information:**

The online version contains supplementary material available at 10.1007/s00774-025-01647-2.

## Introduction

Tendinopathy is characterized as a disorder based on tendonitis or enthesopathy (an acute inflammation) or tendinosis (a chronic inflammation leading to tendon degeneration) at the enthesis region-conditions that may occur alone or in combination [1, 2]. Tendonitis is most common in joints of the extremities, such as the elbow (lateral humeral epicondylitis), heel (Achilles tendonitis), shoulder (rotator cuff tendonitis), and knee (patellar tendonitis), and causes pain during activities such as holding objects, twisting doorknobs and walking. Such injuries interfere not only with work and sports activities, but also with daily activities. Due to the low vascularity of the enthesis region, tissue repair capacity is limited in many of these areas [3]. Thus, if microtears and acute inflammation occur in the enthesis region due to repetitive strain from labor and sports activities or due to aging, and if that strain is repeated without adequate repair, the area often becomes intractable and chronically degenerative, causing long-term pain [4]. Pathologically, tendonitis tissue shows microtears, hypervascularization, and collagen disorganization [5]. One million people per year are reportedly affected by this condition in the United States, and tendonitis leads to social concerns as it decreases work productivity due to pain and other factors [6]. Thus, controlling this disease is mandatory. Since tendons have limited self-repair capacity, it is crucial to adequately treat inflammation in the acute phase to prevent the disease from entering the chronic phase, which frequently induces tendon rupture or degeneration [7]. To date, numerous approaches have been used to treat tendon disorders, including oral and topical NSAIDs, local steroid injections, physical therapy, ultrasound therapy, extracorporeal shock wave therapy, bracing, and surgery [8, 9], but no definitive treatment has yet been established.

Recently, Platelet-Rich-Plasma (PRP) has been used to treat tendon disorders [10]. PRP treatment provides a high concentration of growth factors to the local area to decrease tissue inflammation and promote regeneration and repair of degenerated tissue. However, PRP is currently prepared mainly by blood sampling, which is a minimally invasive procedure, and preparation methods differ among institutions. Moreover, although PRP is usually prepared by blood sampling to administer to a patient immediately before use, it has recently become possible to freeze PRP and store it long-term. However, PRP quality before freezing depends on a patient’s condition at the time of blood collection, and it remains difficult to ensure consistent quality prior to use. It is also difficult to store PRP in a way that maintains its quality, once confirmed. Finally, to date, there are no studies of applying platelets themselves, which are the active components of PRP, to treatment of tendinopathy.

Culture techniques have been devised to differentiate mesenchymal stem cells isolated from subcutaneous adipose tissues (ASCL) in vitro first into megakaryocytes and then into large quantities of platelet-like cells (known as ASCL-PLCs) [11, 12]. Although platelets can also be produced from human induced pluripotent stem cells [13], there are no previous reports of platelet production from adipose stem cells other than ASCL-PLCs. ASCLs meet all the criteria of mesenchymal stem cells, as determined by the International Society for Cell Therapy, and have high safety and uniform properties [14]. ASCL-PLCs can be frozen and thawed and are anticipated to have future medical applications such as platelet replacement transfusion and promotion of wound healing.

In the current study, we hypothesized that ASCL-PLCs can be used to effectively reduce acute inflammation and could serve in tissue repair, similarly to PRP. To test this hypothesis, we established a rat Achilles tendon incision model as a tendonitis model. We report that injections of ASCL-PLCs derived from adipose stem cells into the tendon dissection area effectively antagonized tendon inflammation in the acute phase. We observed no xeno-reactions indicative of rejection following injection of human ASCL-PLCs into wild-type rats. Thus, allogeneic ASCL-PLC transplantation, rather than autologous peripheral blood, could be considered a therapeutic option for acute stage tendinopathy.

## Materials and methods

### Rats

We purchased ten-week-old male Wistar rats (body weight, about 300 g) from Sankyo Labo Service (Tokyo, Japan) and maintained them under specific pathogen-free conditions at animal facilities accredited by the Keio University Institutional Animal Care and Use Committee. All rats were kept under a 12-h light/dark cycle and were fed standard diets. Animal experiments were carried out in accordance with Guidelines and Institutional Guidelines on Animal Experimentation at Keio University.

### ASCL-PLCs

We established ASCLs from adipose-derived mesenchymal stem/stromal cells (ASCs) as previously described [11, 12, 14]. ASCLs represent a more homogeneous population than ASCs and fulfill criteria for mesenchymal stem cells set by the International Society for Cellular Therapy. Cultivation of ASCLs in the presence of MK lineage induction medium led to peak production of platelets within 12 days. ASCL-PLCs or PRPs (each 1.0 × 10^7^ cells) suspended in 50 µl phosphate-buffered saline (PBS), or PBS alone were activated with 10 mM CaCl_2_ and incubated 15 min. In this condition, clots did not form in either ASCL-PLCs or PRPs. Both preparations were then frozen at – 80 ℃ and thawed at room temperature immediately before use.

### Rat Achilles tendonitis model

Tendonitis models were generated under general anesthesia in 10-week-old male rats (n₌48). Control rats underwent sham-surgery via a skin incision in the right hind limb. Model rats received a similarly-sized skin incision on the posterolateral side of the left limb, and the Achilles tendon and flexor tendon wrapped in the paratenon were identified. The paratenon was opened and the Achilles tendon was cut at 3 mm away from the calcaneus without a gap using a scalpel. After the skin was sutured with 5–0 monofilament nylon, either ASCL-PLCs (1.0 × 10^7^ cells/50 µl) or PBS (50 µl) were injected into the left Achilles tendon site using a 27-gauge needle. Bilateral Achilles tendons were harvested three days after surgery for real-time PCR (n₌24), and at 1 (n₌12), 2 (n₌6) or 4 weeks (n₌6) later for histopathology or fluorescent immunohistochemical analysis. All methods were carried out in accordance with the ARRIVE guidelines.

### PRP

To obtain PRP, 30 ml of peripheral blood was collected from healthy adult human volunteers, with approval from the Ethics Committee of our institute (Approval No. 20210093), and blood sampling was carried out with patient consent. Blood was collected into a tube containing citric acid. PRP was obtained by centrifugation (200 × g) at room temperature for 10 min [15], and the PRP (leukocyte-poor; final platelet counts 1.0 × 10^7^/50 µl) was activated with CaCl_2_ and stored at – 80 ℃. All patients provided written informed consent. The study was approved by the Keio University Institutional ethics committee (Approval No. 20210093).

### NIH3T3 cell culture procedures

Adherent NIH3T3 fibroblastic cells, which were maintained in DMEM (Sigma–Aldrich Co.) plus 10% fetal bovine serum (FBS) with penicillin G/streptomycin, were cultured in 96-well plates (1.0 × 10^5^ cells/well) with ASCL-PLC (1.0 × 10^7^ cells/well) with or without both IL-6 (100 ng/ml, R & D Systems) and soluble IL-6 receptor (sIL6-R, 100 ng/ml, R & D Systems). After six hours of cultivation, total RNA was collected to assess IL-6 transcript levels. We also cultured NIH3T3 cells in 96-well plates (1.0 × 10^5^ cells/well) with or without ASCL-PLC (1.0 × 10^5^ cells/well) in the presence or absence of various MAPK inhibitors (2 µM anti-p38, 8 µM anti-JNK or 2 µM anti-ERK) and with or without both IL-6 (100 ng/ml, R & D Systems) and sIL-6R (100 ng/ml, R & D Systems). After six hours of cultivation, mRNA was collected for analysis.

### Realtime PCR analysis

At the time of surgery, the Achilles tendon was cut at 3 mm from the calcaneal attachment site. Three days later, we identified that region under a stereomicroscope, cut out the Achilles tendon with a scalpel, and minced it into small pieces with scissors. RNA was then extracted from those tissues using Trizol reagent (Molecular Research Center, Inc., Cincinnati, OH). Total RNA was similarly prepared from mouse NIH3T3 cell lines described above. In all cases, single-stranded complementary DNAs (cDNAs) were synthesized with reverse transcriptase (FUJIFILM Wako Pure Chemical Corp., Osaka, Japan). Realtime PCR was performed using SYBR Premix ExTaq II (Takara Bio Inc., Otsu, Shiga, Japan) with a DICE Thermal Cycler (Takara Bio Inc.), according to the manufacturer’s instructions. β-actin expression served as an internal control. Primer sequences for real-time PCR were as follows:

*β-actin* (rat)-forward: 5’-TCCTCCCTGGAGAAGAGCTATG-3’.

*β-actin* (rat)-reverse:5’-TGCCACAGGATTCCATACCCAG-3’.

*IL-6* (rat)-forward:5’-TCTCTCCGCAAGAGACTTCCA-3’.

*IL-6* (rat)-reverse: 5’-GGTCTGTTGTGGGTGGTATCC-3’.

*IL-1β* (rat)-forward: 5’-TGTGATGTTCCCATTAGAC-3’.

*IL-1β* (rat)-reverse: 5’-AATACCACTTGTTGGCTTA-3’.

*β-actin* (mouse)-forward: 5’ -TCCTCCCTGGAGAAGAGCTATG-3’.

*β-actin* (mouse)-reverse: 5’-TGCCACAGGATTCCATACCCAG-3’.

*IL-6* (mouse)*-*forward: 5’-GTCCTTAGCCACTCCTTCTG-3’.

*IL-6* (mouse)*-*reverse: 5’ -CAAAGCCAGAGTCCTTCAGAG-3’.

### Histopathology and fluorescent immunohistochemical analysis

Ankle joints including Achilles tendons were removed one, two or four weeks after ASCL-PLC or PBS injection, fixed in 10% neutral-buffered formalin, embedded in paraffin blocks, and cut into 4-μm sections. Ankles were decalcified in 10% EDTA, pH 7.4, before embedding. Hematoxylin and Eosin (HE) staining was performed according to standard procedures. Cells were then observed using a BioRevo microscope and corresponding software (Keyence, Tokyo, Japan). For fluorescent immunohistochemistry assay, after deparaffinization, sections were microwaved 10 min in 10 mM citrate buffer solution (pH 6.0) for antigen retrieval. After blocking one hour with 3% BSA in PBS, sections were stained for 18 h with rabbit anti-mouse IL-6 (1:100 dilution; Abcam) at 4 °C. After PBS washing, sections were stained for one hour with Alexa Fluor 488-conjugated goat anti-rabbit IgG (1:100 dilution; Invitrogen) at room temperature. DAPI (1:1000; (FUJIFILM Wako Pure Chemical Corp., Osaka, Japan) served as a nuclear stain, and cells were observed under a fluorescence microscope (Keyence, Tokyo, Japan).

### Immunoblotting analysis

Whole-cell lysates were prepared from cell cultures using RIPA buffer (1% Tween 20, 0.1% SDS, 150 mM NaCl, 10 mM Tris–HCl (pH 7.4), 0.25 mM phenylmethylsulfonylfluoride, 10 µg /mL aprotinin, 10 µg/mL leupeptin, 1 mM Na3VO4, 5 mM NaF (Sigma-Aldrich Co.)). Equivalent amounts of proteins were separated by SDS-PAGE and transferred to a PVDF (Polyvinylidene Difluoride) membrane (EMD Millipore Corp, Burlington, MA, USA). Membranes were blocked in buffer-containing 10 mM Tris–HCl (pH 7.4), 150 mM NaCl, 0.1% Tween 20, and 5% bovine serum albumin, and incubated with each primary antibody overnight at 4 °C. Primary antibodies used were anti-STAT3 (#4904), anti-pSTAT3 (#9131), (Cell Signaling Technology, Inc., Danvers, MA, USA) or anti-actin antibody (Sigma-Aldrich Co., St Louis, MO, USA). Secondary antibodies were goat anti-mouse IgG (G21040, Thermo Fisher Scientific, Waltham, Massachusetts, USA) and goat anti-rabbit IgG (G21234, Thermo Fisher Scientific). Membranes were then incubated using the appropriate secondary antibodies, and the immune complexes visualized using the ECL Western Blotting Analysis System (GE Healthcare, Tokyo, Japan). Image J v. 1.51 (the National Institutes of Health, Bethesda, MD) was used to quantify each band.

### Statistical analysis

Results are expressed as means ± s.d. Statistical significance of differences between groups was evaluated using unpaired two-tailed Student’s t-test or ANOVA (^†^ *P* < 0.10; * P < 0.05; ** P < 0.01; *** P < 0.001; NS, not significant, throughout the paper).

## Results

### ASCL-PLC treatment inhibits IL-6 transcript expression and Stat3 phosphorylation in NIH3T3 cells stimulated with IL-6/sIL-6R

**Fig. 1:**
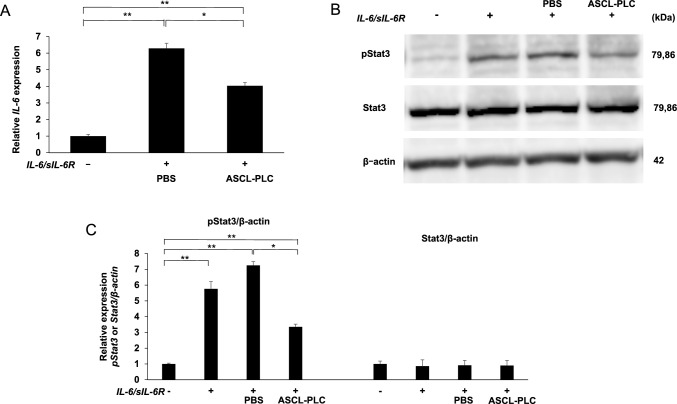
ASCL-PLC inhibits IL-6 expression and Stat3 phosphorylation in NIH3T3 cells stimulated with IL-6/sIL-6R. We stimulated NIH3T3 cells with IL-6/sIL6-R (100 ng/ml each) with or without ASCL-PLC (1.0 × 10^7^/50 µl). After six hours of treatment, we analyzed IL-6 transcript levels (**A**). Data in indicated groups represent mean IL-6 transcript levels normalized to β-actin ± SD relative to control (IL-6/sIL6-R (-)). We also stimulated NIH3T3 cells similarly, cell lysates were prepared for western blot with total and phospho- Stat3. Actin served as an internal control (**B**). Intensity levels of pStat3 and total Stat3 bands were quantified using image J and shown as mean band intensity ± SD (**C**)

IL-6 expression is reportedly induced by auto-amplification of IL-6/soluble IL-6 receptor (sIL-6R) signaling [16], which in turn phosphorylates and activates the transcription factor Stat3 [17]. To evaluate ASCL-PLC effects on this pathway, we stimulated NIH3T3 cells with IL-6/sIL6-R (100 ng/ml each) with or without ASCL-PLCs (1.0 × 10^7^ /50 µl). 6 h later, we analyzed IL-6 transcript levels by real-time PCR. Relative to untreated controls, IL-6 transcript expression induced by IL-6/sIL-6R stimulation was significantly inhibited by ASCL-PLC treatment (Fig. 1 A). In separate analysis, we also stimulated NIH3T3 cells in the same methods, prepared cell lysates ten minutes later, and performed western blot analysis with antibodies recognizing total and phospho- Stat3. That analysis revealed that, relative to PBS control cells, Stat3 phosphorylation induced by IL-6/sIL-6R was significantly inhibited by ASCL-PLC treatment. (Fig. 1 B and 1 C).

### ASCL-PLC treatment inhibits IL-6 expression induced by IL-6/sIL-6R in NIH3T3 cells via the p38 pathway

**Fig. 2:**
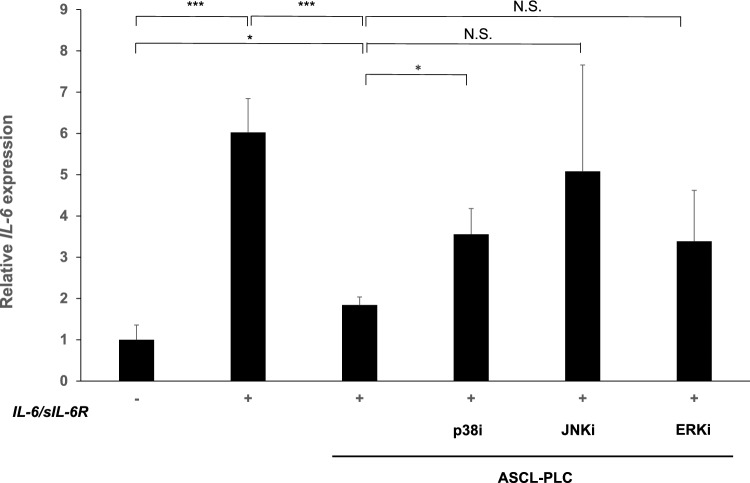
ASCL-PLC inhibits IL-6 expression induced by IL-6/sIL-6R in NIH3T3 cells via the p38 pathway. NIH3T3 cells were stimulated with IL-6/sIL6-R (100 ng/ml each) first, treated with or without ASCL-PLC (1.0 × 10^7^/50 µl). Then ASCL-PLC groups were treated with one of three MAPK inhibitors, p38i (2 µM), JNKi (8 µM) or ERKi (2 µM). Six hours later, IL-6 expression was analyzed. Data represents mean IL-6 transcript levels normalized to β-actin ± SD in indicated conditions relative to control (IL-6/sIL6-R (-))

We next analyzed signaling underlying inhibition of IL-6 expression mediated by ASCL-PLCs in NIH3T3 cells. To do so, we stimulated NIH3T3 cells ten minutes with IL-6/sIL-6R (100 ng/ml each) then treated with or without ASCL-PLCs (1.0 × 10^7^ /50 µl). Then ASCL-PLC groups were treated with MAPK inhibitors, p38i (2 µM), JNKi (8 µM) or ERKi (2 µM). Expression of IL-6 transcripts induced by IL-6/sIL-6R was significantly inhibited by ASCL-PLC treatment; however, that inhibition was significantly blocked by a co-treatment with the p38 inhibitor (Fig. 2).

### Induction of acute inflammation at the incision site of rat Achilles tendon

**Fig. 3:**
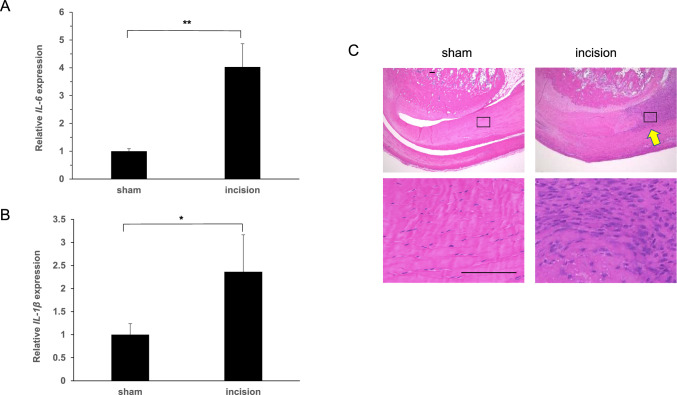
Achilles tendon incision promotes inflammation at tenotomy sites in rats. The left Achilles tendon of 10-week-old male rats were incised at the enthesis of calcaneus. The right side was sham operated. Three days later, surgical sites were harvested and expressions of IL-6 and IL-1β was analyzed. Data represent mean expression of IL-6 (**A**) or IL-1β (**B**) relative to β-actin ± SD (each n = 3). One week later, both lower ankle joints containing the Achilles tendon were removed and tissue sections were stained with HE and observed under a microscope (**C**) (each n = 3). Scale bar = 100 μm. Arrow indicates inflammatory cell infiltration at the Achilles tendon incision site

To establish the tendinopathy model (precisely, tendonitis model), we made an incision in the left Achilles tendon of rats at the calcaneal enthesis, and the right side was received only skin incision (sham-operated) at the same site. 3 days later, we evaluated expression of the inflammatory cytokines IL-6 and IL-1β in the Achilles tendon calcaneal enthesis of both groups by real-time PCR. Both IL-6 and IL-1β expression was significantly elevated at the incision compared to the sham side (Fig. 3 A and 3 B). Moreover, histological HE stains revealed inflammatory cell infiltration at the incision area relative to the sham side, suggesting that acute inflammation had been induced by the incision (Fig. 3 C).

### ASCL-PLC treatment inhibits acute inflammation induced by Achilles tendon incision in rats

**Fig. 4:**
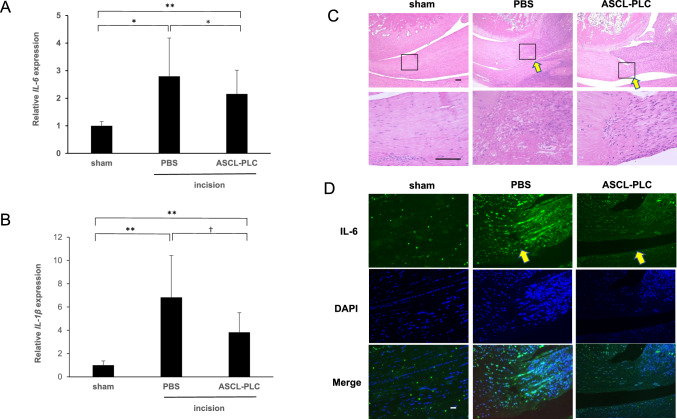
ASCL-PLC suppresses inflammatory cytokine expression at tenotomy sites in rats. We injected either ASCL-PLCs (1.0 × 10^7^ cells) or control PBS (50 µl each) into the rat left Achilles tendon incision sites. The sham site only received skin incision. Three days later, we evaluated expressions of IL-6 and IL-1β at surgical sites. Data represent mean expression of IL-6 (**A**) or IL-1β (**B**) relative to β-actin ± SD (each n = 6). One week later, Achilles tendon enthesis region were harvested, stained with HE and observed under a microscope (**C**) (each n = 3). Tissue sections were also stained with rabbit anti-mouse IL-6 followed by Alexa Fluor 488-conjugated goat anti-rabbit Igs’ (green), as well as the nuclear marker DAPI (blue), and observed under a microscope (**D**). All Scale bar = 100 μm. Arrows indicate Achilles tendon tenotomy site

To evaluate potential anti-inflammatory effects of ASCL-PLC treatment, we injected either ASCL-PLCs (1.0 × 10^7^ cells/50 µl) or control PBS (50 µl) into the rat Achilles tendon incision site. The right side was sham operated. Then, three days later, we evaluated IL-6 and IL-1β expression in the Achilles tendon enthesis area of both incision and sham-operated groups by real-time PCR. In control PBS groups, both IL-6 and IL-1β were significantly upregulated at the incision site compared to the sham control side. Importantly, expression of both IL-6 and IL-1β was significantly suppressed at the incision site in ASCL-PLC groups relative to the control PBS (Fig. 4 A and 4 B).

In separate analysis we performed the same procedures but sacrificed ASCL-PLC-treated rats or PBS-treated controls one week after treatment and performed HE and IL-6 staining of the Achilles tendon enthesis region. Xeno-reaction was reportedly induced various characteristic tissue reactions including tissue hemorrhage [18]. We observed no xeno-reactions in rats injected with human ASCL-PLCs, based on HE staining. Moreover, at one week after surgery, inflammatory cell infiltration at the incision site was decreased in the ASCL-PLC relative to the PBS group, based on HE staining (Fig. 4 C). Immunofluorescence analysis also indicated that IL-6 protein expression at the Achilles tendon incision site decreased in ASCL-PLC versus PBS control samples (Fig. 4 D). Moreover, 2 weeks after surgery, the ASCL-PLC group showed greater tissue repair based on HE-staining than did the control group, and the Achilles tendon became more properly oriented. By 4 weeks after surgery, differences between the two groups became less apparent, and spontaneous recovery was seen at that time point in operated rats not treated with ASCL-PLC injection (Fig. S1). Similarly, when tissue repair was evaluated semi-quantitatively based on the Bonar score, ASCL-PLC treatment significantly accelerated tissue repair relative to PBS by 1 week after the incision, but by 4 weeks repair was comparable in both the ASCL-PLC and PBS groups (Fig. S2 and S3 A-E) [19].

### ASCL-PLC and conventional PRP inhibit IL-6 expression induced by Achilles tendon incision in rats

**Fig. 5:**
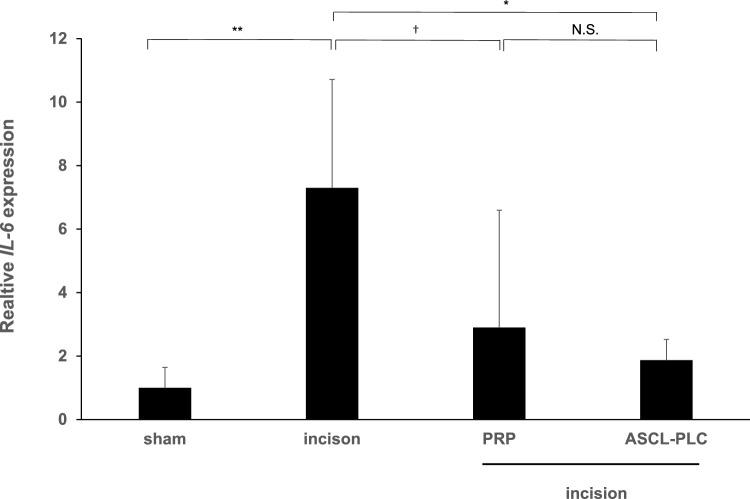
ASCL-PLC and conventional PRP exert comparable anti-inflammatory effects on Achilles tendon tenotomy sites in rats. We administered either human peripheral blood platelet-rich plasma (PRP, leukocyte-poor; final platelet count 1.0 × 10^7^ /50 µl) or ASCL-PLCs (1.0 × 10^7^ /50 µl) to the rat Achilles tendon incision sites. After three days, surgical sites were harvested, and IL-6 expression was analyzed. Data represent mean IL-6 transcript levels normalized to β-actin ± SD in indicated conditions relative to control (IL-6/sIL6-R (-)) (each n = 3)

Finally, to compare effects of PRP and ASCL-PLC on IL-6 expression, we administered either human peripheral blood platelet-rich plasma (PRP, leukocyte-poor; final platelet count (1.0 × 10^7^/50 µl) or ASCL-PLCs (1.0 × 10^7^/50 µl) to the rat Achilles tendon injury model immediately after Achilles tendon incision (each n = 3). Three days later, we harvested Achilles tendon tissues at the incision site, we performed real-time PCR to detect IL-6 transcripts. As anticipated, IL-6 transcripts were significantly elevated in incision versus shame-operated tissues. Moreover, ASCL-PLC treatment significantly suppressed IL-6 transcript expression at the Achilles tendon incision sites (Fig. 5).

## Discussion

Tendinopathy, including lateral epicondylitis and Achilles tendonitis, is a disorder frequently seen in both athletes and the elderly. Acute tendonitis is thought to be caused by repeated tendon loading, and pain due to inflammatory cytokine activity interfering with activities of daily living and with work and sports activities [2]. Since acute inflammation, if not adequately attenuated, leads to advanced and intractable chronic degeneration (tendinosis) [4], it is important to control the pathophysiology of tendon disorders during the acute inflammatory phase before the transition to tendinosis. Moreover, tendon tissues are known to have limited capacity for repair after injury [20]. In the present study, we found that treatment with human ASCL-PLCs significantly suppressed inflammation and promoted tissue repair in a rat Achilles tendon incision model of acute tendonitis likely by inhibiting IL-6 expression and blocking p38 MAPK-dependent Stat3 activation. We also showed that in a rat Achilles tendon incision model, ASCL-PLC treatment significantly accelerated tissue repair relative to PBS-treated samples by 2 weeks post-incision, but that PBS controls and ASCL-PLC-treated samples showed similar levels of tissue repair by 4 weeks. These observations suggest that ASCL-PLC treatment shortens the tissue repair period.

Tendinopathy models such as injury and collagenase models have been established for the Achilles tendon in mice and rats [21]. However, we employed an incision model to mimic the micro-rupture and subsequent inflammation induced in tendons during tendinopathy as an acute inflammation model. Various treatments for tendon disorders have been used such as local steroid injections. While such injections are effective against tendonitis, repeated steroid administration is known to weaken tendon strength [22, 23]. Recently, the usefulness of PRP therapy for tendon disorders has been reported. PRPs have anti-inflammatory and tissue repair-promoting effects based on local administration of high concentrations of the various growth factors contained in platelets [24–26]. At present, PRPs are mainly produced from autologous blood, and their method of preparation and quality vary among institutions. Although it is now technically feasible to freeze and store PRP, it is often possible to obtain only a small amount of PRP from collected blood, and the quality of the preparation depends on a patient’s condition at the time of blood sampling [26–28]. Also, the presence or absence of leukocytes is known to alter their efficacy. For example, leukocyte-rich-PRP, which as its name suggests contains a greater quantity of leukocytes, has been shown to promote inflammation and catabolism at administration sites and contains higher levels of matrix metalloproteinases (MMPs) [29, 30]. By contrast, leukocyte-poor PRP (Pure-PRP), which we used in the analysis shown on Fig. 5, contains fewer leukocytes and is reportedly more effective in treating early tendon disorders, soft tissue inflammation and knee osteoarthritis [29]. Nonetheless, Pure-PRP contains a small number of leukocytes, and the amount that can be obtained from a single blood draw is small.

As an alternative approach, in this study we used ASCL-PLCs, which are platelets differentiated from a human mesenchymal stem cell/stromal cell line (ASCL) derived from subcutaneous adipose tissues. This preparation has several advantages: it is relatively homogeneous, can be freeze-thawed, does not require a blood draw from a patient, and can be mass-produced in a consistent manner [11, 12, 14]. In our analysis, ASCL-PLC preparations were stored at -80 °C and brought to room temperature before use, and even when stored for up to ~ 6 months, retained their anti-inflammatory effect (Figs. 1, 2, 4, S1 and 5). Although several facilities use cryopreserved PRP in clinical practice, maintaining its homogeneity by drawing a small amount of blood from each patient remains a challenge. Thus, ASCL-PLCs could serve as a cryopreservable alternative. ASCL-PLCs have no nuclei, and we evaluated their quality after cryopreservation by their ability to respond to stimuli by releasing growth factors. We confirmed that ASCL-PLCs were responsive to stimuli after 9 months of cryopreservation (data not shown). While PRPs are derived from hematopoietic stem cells (HSCs), ASCL-PLCs are induced from mesenchymal stem cells and thus have both mesenchymal stem cell and HSC characteristics. In fact, it was previously shown that ASCL-PLCs express CD90, which is not expressed in PRPs [12]. CD90 reportedly functions in cell adhesion [31], and therefore, ASCL-PLCs expressing CD90 may be more likely to remain in injected tissues than PRPs. Furthermore, here, human-derived ASCL-PLCs did not induce rejection in xeno-transplanted wild-type rats and promoted anti-inflammatory effects. These observations suggest the possibility of allogeneic transplantation in humans, although further studies are needed in this regard.

PRP reportedly has anti-inflammatory effects in cases of tendon disorders, osteoarthritis and skin disorders [32]. IL-6 protein has a longer half-life than TNF-α and IL-1β, and thus, IL-6 is useful in assessing inflammation and determining efficacy of a particular therapy. During inflammation, IL-6 is upregulated by Jak/Stat pathways [17, 33], and it was previously reported that MAPKs interfere with the Stat pathway [34]. Among MAPK pathways, activation of ERK1/2 reportedly functions to inhibit Stat3 activation in myeloid or oral cancer cells [35, 36]. In contrast, our present study suggests that ASCL-PLC treatment inhibits Stat3 phosphorylation by activating p38 and that cross-talk between MAPK and Stat3 pathways is cell type-specific.

Taken together, we conclude that ASCL-PLC has advantages over existing autologous PRP in that; it does not require blood sampling, does not contain leukocyte components, is more homogeneous than PRP, can be made in large quantities, its efficacy can be verified in advance, it can be frozen and stored while maintaining its anti-inflammatory effect, and it can be dissolved and used when needed. PRP manufacturing costs vary depending on the method or the type of manufacturing kit used. On the other hand, ASCL-PLC can be mass-produced and stored, but manufacturing costs vary depending on the manufacturing scale. Therefore, at present, simple comparisons of PRP and ASCL-PLC manufacturing costs remain difficult to make. Since ASCL-PLCs could be used for xenografts and platelet products are routinely used in clinical practice for allogeneic transplantation, it represents as a future clinical application of allografts to treat tendinopathy.

## Supplementary Information

Below is the link to the electronic supplementary material.

Supplementary file1 (PDF 3402 KB)
