# Supplementary material for: Platelet-like cells differentiated from adipose-derived mesenchymal stem cells inhibit acute inflammation of tendinopathy in rats
Source: J Bone Miner Metab. 2025 Oct 3;44(1):18–27. doi: 10.1007/s00774-025-01647-2 (PMC12890972; doi:10.1007/s00774-025-01647-2)
Supplement: Supplementary file 1 — Supplementary file1 (PDF 3402 KB) [file 774_2025_1647_MOESM1_ESM.pdf]

Supplementary Figure Legend

Supplementary Figure 1. ASCL-PLC treatment promotes tissue repair at tenotomy sites in rats.

To create our model, we performed tenotomy of the rat left Achilles tendon and then injected either ASCL-PLCs (1.0 × 10^7 cells) or control PBS (50μl each) into incision sites. Sham-operated rats received only a skin incision without any treatment. One, two or four weeks later, Achilles tendon enthesis regions were harvested, stained with HE and observed under a microscope. N=3 each, 1 week after operation; N=6 each, 2 and 4 weeks after operation. Scale bar=100μm. Higher magnification images of regions boxed in lower magnification images (at left) are shown in corresponding images at right.

Supplementary Figure 2. ASCL-PLC treatment shortens the repair period at tenotomy sites in rats.

Tenotomy was performed in the rat left Achilles tendon and then incision sites were injected either ASCL-PLCs (1.0 × 10^7 cells) or control PBS (50μl each). One, two or four weeks later, Achilles tendon enthesis regions were harvested, stained with Alcian blue (A), observed under a microscope, and the Bonar score was calculated (B). Data represent mean Bonar score at the indicated time point ±SD (N=3 each, 1 week after operation; N=6 each, 2 and 4 weeks after operation). Scale bar=100μm.

Supplementary Figure 3. Analysis of each Bonar score components in Achilles tendons.

Tenotomy was performed in the rat left Achilles tendon and then incision sites were injected either ASCL-PLCs (1.0 × 10^7 cells) or control PBS (50μl each). One, two or four

weeks later, Achilles tendon enthesis regions were harvested, stained with HE or Alcian blue. Bonar score components in rat Achilles tendons treated with ASCL-PLC or PBS are shown. The Bonar score is semi-quantitative assessment, which is calculated as the sum of five histological parameters- cell morphology (A), collagen arrangement (B), cellularity (C), vascularity (D), and ground substance (E)-evaluated via HE and Alcian blue staining. Each was scored on a scale 0 (normal) to 3 (severely abnormal). Significant differences were observed at 2 weeks in collagen arrangement and ground substance. (N=3 each, 1 week after operation; N=6 each, 2 and 4 weeks after operation).

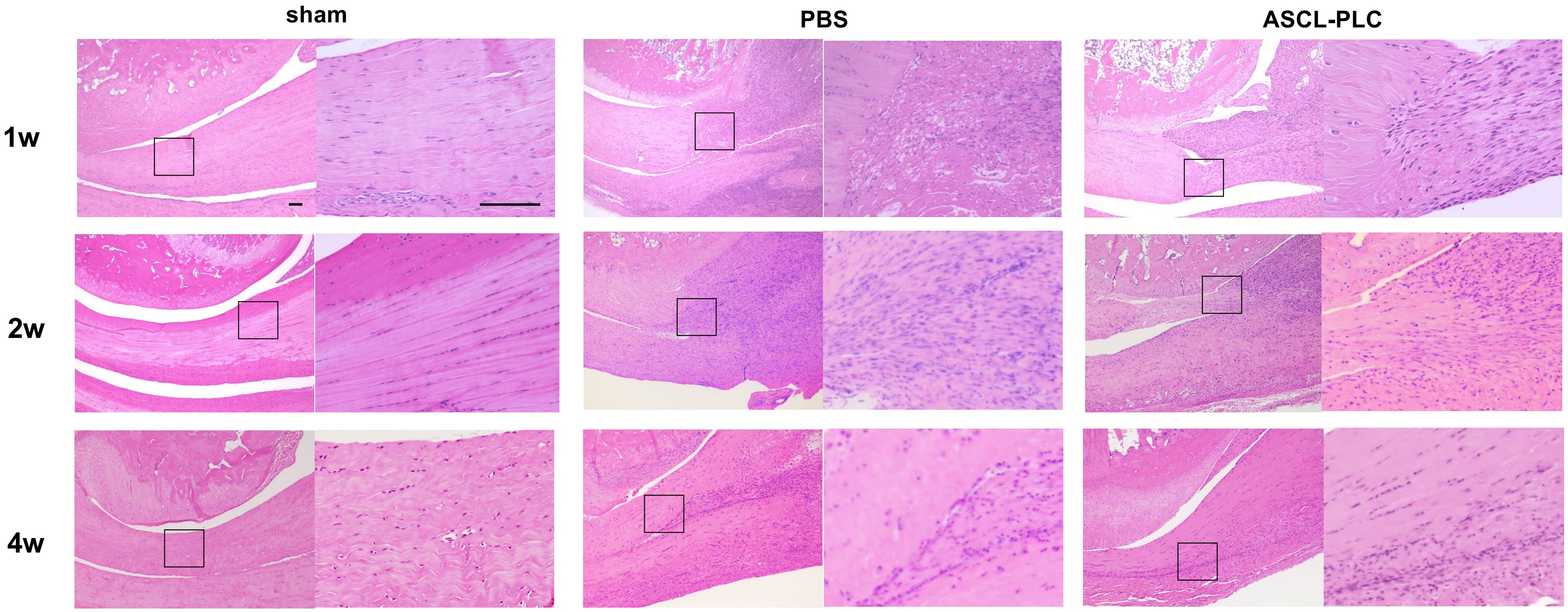

Supplementary Figure 1

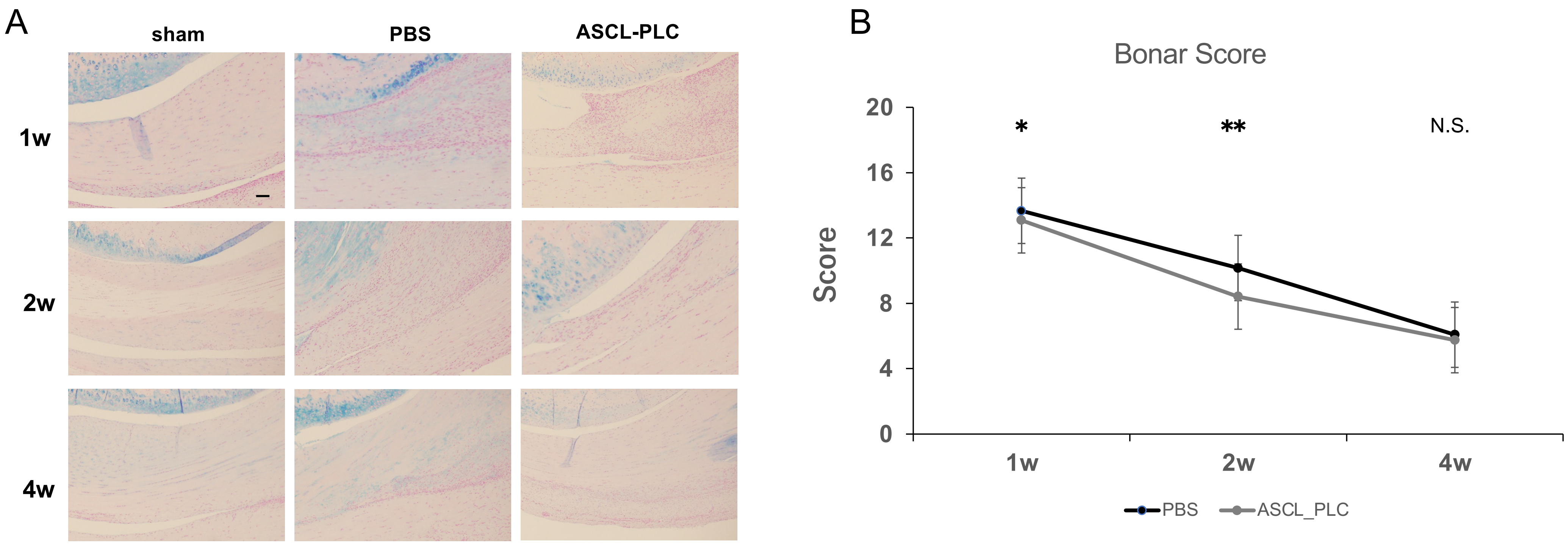

Supplementary Figure 2

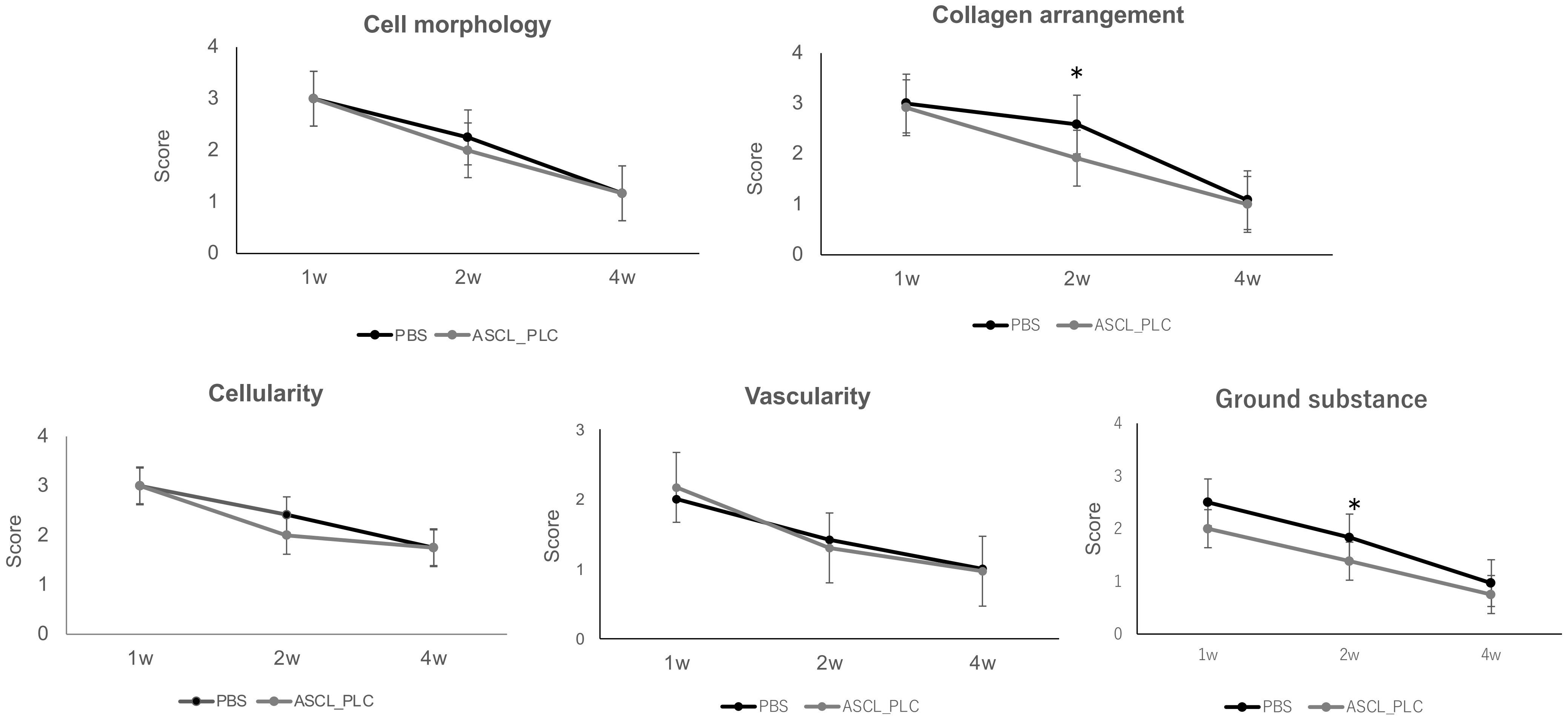

Supplementary Figure 3
